# The temperature of growth and sporulation modulates the efficiency of spore-display in *Bacillus subtili*s

**DOI:** 10.1186/s12934-020-01446-6

**Published:** 2020-10-01

**Authors:** Claudia Petrillo, Stefany Castaldi, Mariamichela Lanzilli, Anella Saggese, Giuliana Donadio, Loredana Baccigalupi, Ezio Ricca, Rachele Isticato

**Affiliations:** 1 Department of Biology, Federico II University complesso universitario di Monte Sant’ Angelo via Cinthia, 80126 Napoli, Italy; 2 Department of Molecular Medicine and Medical Biotechnology, Federico II University of Naples, Napoli, Italy; 3 Present Address: Institute of Biomolecular Chemistry, National Research Council of Italy, Pozzuoli (Naples), Italy; 4 Present Address: Department of Medicine, Surgery and Dentistry “Scuola Medica Salernitana”, University of Salerno, Fisciano (SA), Italy

**Keywords:** Display platform, Mucosal vaccines, *Bacillus subtilis*, Probiotics

## Abstract

**Background:**

Bacterial spores displaying heterologous antigens or enzymes have long been proposed as mucosal vaccines, functionalized probiotics or biocatalysts. Two main strategies have been developed to display heterologous molecules on the surface of *Bacillus subtilis* spores: (i) a recombinant approach, based on the construction of a gene fusion between a gene coding for a coat protein (carrier) and DNA coding for the protein to be displayed, and (ii) a non-recombinant approach, based on the spontaneous and stable adsorption of heterologous molecules on the spore surface. Both systems have advantages and drawbacks and the selection of one or the other depends on the protein to be displayed and on the final use of the activated spore. It has been recently shown that *B. subtilis* builds structurally and functionally different spores when grown at different temperatures; based on this finding *B. subtilis* spores prepared at 25, 37 or 42 °C were compared for their efficiency in displaying various model proteins by either the recombinant or the non-recombinant approach.

**Results:**

Immune- and fluorescence-based assays were used to analyze the display of several model proteins on spores prepared at 25, 37 or 42 °C. Recombinant spores displayed different amounts of the same fusion protein in response to the temperature of spore production. In spores simultaneously displaying two fusion proteins, each of them was differentially displayed at the various temperatures. The display by the non-recombinant approach was only modestly affected by the temperature of spore production, with spores prepared at 37 or 42 °C slightly more efficient than 25 °C spores in adsorbing at least some of the model proteins tested.

**Conclusion:**

Our results indicate that the temperature of spore production allows control of the display of heterologous proteins on spores and, therefore, that the spore-display strategy can be optimized for the specific final use of the activated spores by selecting the display approach, the carrier protein and the temperature of spore production.

## Introduction

Endospores (spores) are quiescent cell forms produced by over 1000 bacterial species when the environmental conditions do not allow cell growth to continue [1]. In the spore form, these bacterial species can survive conditions, such as the prolonged absence of water and nutrients, the exposure to extremes of temperature and pH, to UV irradiations and to toxic chemicals, that would be lethal for other cell forms [2]. Although metabolically quiescent, the spore is able to sense the environment and respond to conditions that allow cell growth by germinating and generating a new vegetative cell [3]. Spore germination and resistance are in part due to the peculiar structure of the spore, that has been studied in detail in *Bacillus subtilis,* the model system for spore formers [2, 4]. In *B. subtilis,* spores are formed by a partially dehydrated cytoplasm (core) surrounded by several protective layers: the thick peptidoglycan-like cortex, the multilayered, proteinaceous coat and the crust, the outermost layer formed of proteins and glycans [4]. In some species, including *B. anthracis, B. cereus* and *B. megaterium*, the outermost layer of the coat is the exosporium, a protective shell mainly made of glycoproteins [4].

The rigidity and compactness of the spore suggested the possibility of using this unusual cell as a platform to display heterologous proteins [5]. In a *proof-of-concept* work, the spore coat protein CotB of *B. subtilis* was used as a carrier to display the C fragment of the tetanus toxin (TTFC) of *Clostridium tetani* on the spore surface [5]. To this aim a genetic system was developed to generate gene fusions between the *cotB* gene and DNA coding for TTFC and to allow expression of the fusion during sporulation [5]. The mucosal administration of recombinant spores displaying TTFC was then shown protective against a challenge with the tetanus toxin and able to induce humoral and cellular immune responses [6, 7]. Over the years, the same approach has been used with other coat proteins as carriers and a variety of other heterologous proteins [8]. However, this display system has the drawback of generating recombinant spores, that in case of a field use could raise safety concerns [9]. To overcome this problem a non-recombinant display system based on the spontaneous and stable adsorption of heterologous proteins to bacterial spores has been also developed [10, 11]. Antigens and enzymes have been efficiently and stably adsorbed to spores [12, 13] and it has been proposed that the adsorption is due to the negative electric charge and the relative hydrophobicity of the spore surface [10, 14]. In addition, studies with *B. subtilis* and *B. megaterium* indicated that some proteins were able to infiltrate through "pores" of the outermost spore coat layers and localize in the inner coat of *B. subtilis* spores [15] or in the interspace between the exosporium and the outer coat in *B. megaterium* spores [16, 17].

The spore-display system by both the recombinant or non-recombinant approach, provides several advantages with respect to other display systems, such as a high stability even after a prolonged storage, the possibility of displaying large, multimeric proteins and the safety for a human use, demonstrated by the wide use of spores of some species as probiotics [18, 19]. Based on these, the activated spore has been proposed as a mucosal delivery system, as a vaccine vehicle, as a functionalized probiotic and as a platform to display enzymes [8, 20].

Both approaches are quite efficient, and it has been estimated that up to 3.0 × 10^3^ heterologous molecules can be displayed by each recombinant spore of *B. subtilis* [8, 21]. The efficiency of the non-recombinant approach can be higher than that measured for the recombinant system and depends on the heterologous protein and the *Bacillus* species used [12, 15, 16, 20]. In spite of the efficiency of these systems, the possibility to increase and/or control the number of heterologous proteins presented on the spore is an important achievement for the full exploitation of this biotechnology tool. In the case of a use as a vaccine vehicle, for example, an increased efficiency of display results in a higher dose of antigen delivered or reduced amounts of spores needed for the immunization.

Based on a recent report showing that *B. subtilis* builds spores with different structure when grown at 25, 37 or 42 °C [22], we investigated whether the efficiency of spore-display by both recombinant and non-recombinant approaches could be modulated by modifying the temperature of spore production.

## Results and discussion

### Effects of the temperature on the recombinant display system

CotB, CotC and CotG are abundant coat proteins widely used as carriers to display heterologous proteins on the spore surface [8]. All three proteins have been recently found differentially represented in spores produced at 25, 37 or 42 °C, with CotB and CotG more abundant in spores prepared at 25 °C and CotC more abundant in 42 °C spores [22]. We used isogenic *B. subtilis* strains carrying DNA coding for the model antigen TTFC (*tetC*) fused to the gene coding for either CotB (*cotB*) [5] or CotC (*cotC*) [23] to evaluate the effect of the sporulation temperature on the fusion proteins. Spores of strains RH103 (*cotB::tetC*) and RH114 (*cotC::tetC*) were produced at 25, 37 and 42 °C and purified, as previously reported [22]. Surface proteins were extracted from RH103 and RH115 spores by the SDS-DTT or NaOH treatments, respectively and used for western blotting analysis with anti-CotB [5] or anti-CotC [23] antibodies.

**Fig. 1:**
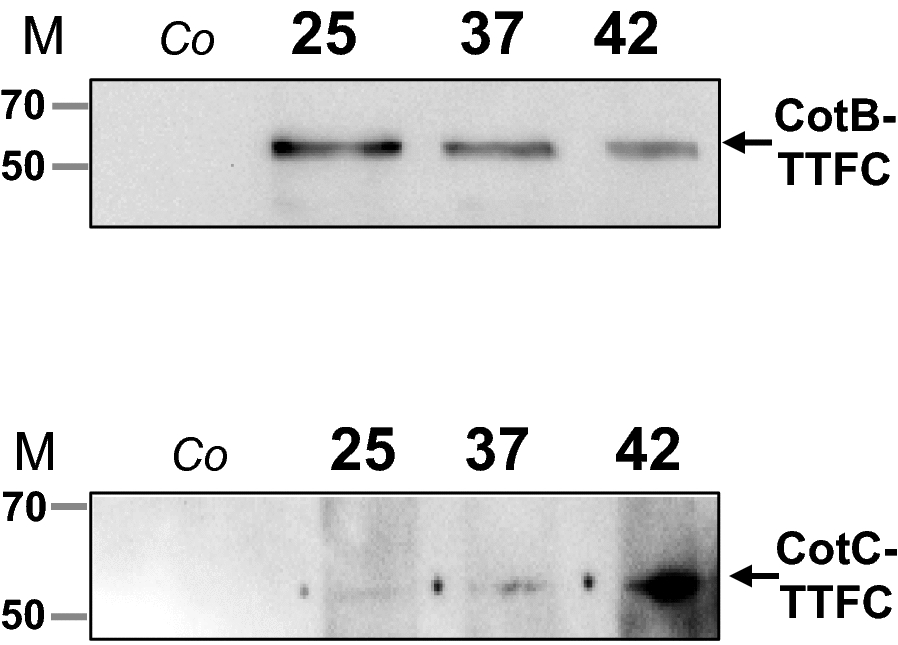
Western blotting analysis of coat proteins. Purified spores of strains carrying the *cotB::tetC* (upper panel) or *cotC::tetC* (lower panel) were produced at the three temperatures and used to extract coat proteins by SDS-DTT (upper panel) or NaOH (lower panel) treatment as previously reported [22]. Proteins were reacted with anti-CotB (upper panel) or anti-CotC (lower panel) antibody. Control lanes (Co) were loaded with proteins extracted from spores of strains carrying either a *cotB* (upper panel) or a *cotC* (lower panel) null mutation

As shown in Fig. 1, specific CotB-TTFC (upper panel) and CotC-TTFC (lower panel) signals were observed in all the samples but not in the negative controls, revealing that the temperature did not affect the self-assembly of the heterologous proteins around the spores. Moreover, we observed that the fusion protein CotB-TTFC was more represented in 25 °C spores than in 37 or 42 °C spores (upper panel), while the fusion CotC-TTFC showed the opposite trend (lower panel).

**Fig. 2:**
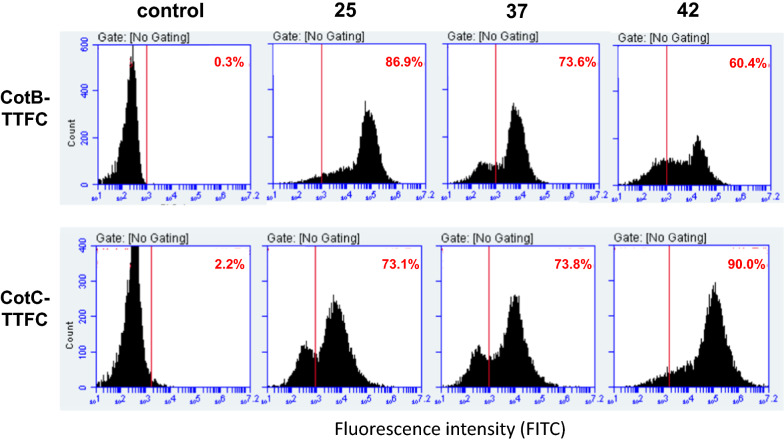
Flow cytometry analysis of spores of strains carrying the *cotB::tetC* or *cotC::tetC* gene fusion. Spores were produced at 25, 37 or 42 °C and reacted with anti-TTFC antibody, then with FITC-conjugated secondary antibody. The analysis was then performed on the entire spore population (ungated). In each panel is indicated the percentage of positive events above the fluorescent intensity threshold of 1 × 10^3^ (red line). As control experiment spores of an isogenic wild type strain (PY79) not containing any gene fusion were analyzed

A flow cytometry approach was used to confirm and quantify the differences in the display of CotB-TTFC and CotC-TTFC at the various temperatures and evaluate their surface exposure. Spores of strains RH103 and RH114 were reacted with anti-TTFC [7] antibodies, then with fluorescently labeled secondary antibody and analyzed by flow cytometry as previously reported [24]. The threshold of positive events was set at 1 × 10^3^ fluorescence intensity and the percentages of fluorescent events for each temperature are indicated in red in each panel. The flow cytometry analysis indicated that CotB-TTFC was displayed with the highest efficiency in spores prepared at 25 °C (86.9% positive events) and that such efficiency decreased in 37 and 42 °C spores (Fig. 2). The efficiency of display was opposite with CotC-TTFC with the highest levels observed with 42 °C spores (90.0% of positive events) and lower levels with 37 and 25 °C spores (Fig. 2). In addition, the fluorescent intensity peak for CotB-TTFC was tenfold higher at 25 °C than at 42 °C while for CotC-TTFC was tenfold higher at 42 °C than at 25 °C, suggesting that the sporulation temperature affected not only the amount of assembled heterologous proteins but also their surface display.

**Fig. 3:**
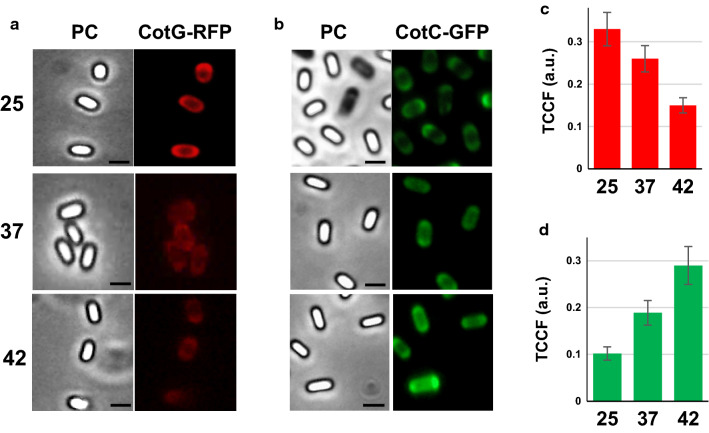
Fluorescence microscopy analysis of spores displaying CotG-RFP or CotC-GFP. **a**–**c** Spores of strain RH296, carrying the *cotG::rfp* fusion (**a**) and of strain RH238, carrying the *cotC::gfp* fusion (**b**) were produced at 25, 37 or 42 °C and observed with a fluorescent microscope. The exposure time was the same for all samples with the same reporter gene. Phase contrast (PC), red-fluorescence (CotG-RFP) or green-fluorescence (CotC-GFP) microscopy images of representative spores are reported. Scale bar 1 μm. **b**–**d** Quantitative analysis of the fluorescence of more than 300 spores as in panels **a**–**c**, performed with the ImageJ software, as previously reported [22]. The Y-axis describes the Total corrected cellular fluorescence (TCCF) value

Results of Figs. 1, 2 indicated, respectively, the amounts of fusion proteins extracted and exposed on the spore surface but did not allow to exclude that other amounts of each fusion were actually present (but not extracted or not exposed) on spores produced at different temperatures. To address this issue, we used different isogenic strains of *B. subtilis* RH238, carrying the Green Fluorescent Protein (GFP) fused to CotC [23], and RH296, carrying the Red Fluorescent Protein (RFP) fused to CotG [22]. A fluorescence microscopy analysis on spores prepared at 25, 37 or 42 °C and the quantification of the fluorescence signals performed by the ImageJ software, as previously reported [24], indicated that the CotG-based fusion was more abundant at 25 °C, less abundant at 37 °C and almost undetectable at 42 °C while the CotC-based fusion showed an opposite pattern (Fig. 3).

Results of Fig. 3, confirming results of Figs. 1,2, allow to conclude that the CotB- and CotG-based fusions are efficiently displayed when spores are produced at 25 °C, while CotC-based fusions are better displayed when spores are produced at 42 °C and, therefore, that is possible to modulate the amount and the surface exposure of fusion proteins displayed on the spore by changing the temperature of spore production on the base of the carrier protein used for the display.

### Effects of the temperature on recombinant spores displaying two fusion proteins

**Fig. 4:**
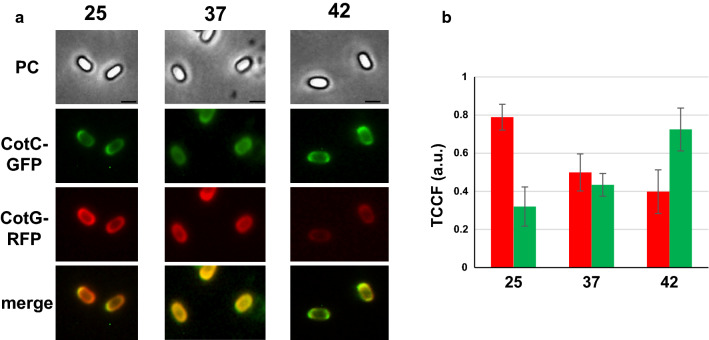
Fluorescence microscopy analysis of spores displaying simultaneously CotG-RFP and CotC-GFP. **a** Spores of strain RH406, carrying the *cotG::rfp* and *cotC::gfp* fusions were produced at the three temperatures and observed with a fluorescent microscope. The exposure time was the same for all samples. Phase contrast (PC), red- fluorescence (CotG-RFP) and green-fluorescence (CotC-GFP) microscopy and merge of the fluorescence images of representative spores are reported. **b** Quantitative analysis of the fluorescence of 200 spores as in panel a, performed with the ImageJ software, as previously reported [22]. The Y-axis describes the Total corrected cellular fluorescence (TCCF) value

An extension of the recombinant spore-display technology is the use of spores carrying more than one heterologous protein. By chromosomal DNA-mediated transformation [25], the gene fusion carried by strains RH238 (*cotC::gfp*) was moved into strain RH296 (*cotG::rfp*) obtaining strain RH406 that carried both fusions. As shown in Fig. 4, spores of strain RH406 presented both fluorescent proteins on their surfaces in similar amounts when spores were grown at 37 °C. When spores were produced at 25 °C the red fluorescent signal (CotG-RFP) was more abundant than the green one (CotC-GFP) that was instead predominant when spores were grown at 42 °C.

Results of Fig. 4 highlight an important improvement for the spore-display technology, showing that it is possible to produce spores that simultaneously display two heterologous proteins and to control which displayed protein has to be more abundantly represented by selecting the temperature of spore production.

### Effects of the temperature on the non-recombinant display system

**Fig. 5:**
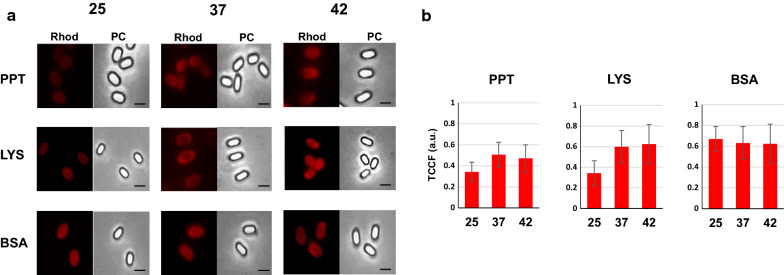
Fluorescence microscopy of spores adsorbed with rhodamine-labeled PPT, LYS or BSA. **a** Fluorescence of PPT-Rd, LYS-Rd or BSA-Rd upon adsorption to wild type spores grown at 25, 37 or 42 °C. The same microscopy field was observed by phase contrast (PC) and fluorescence microscopy (Rhod). The exposure time was the same for all samples. **b** Quantitative analysis of the fluorescence of 300 spores as in panel a, performed with the ImageJ software, as previously reported [22]. The Y-axis describes the Total corrected cellular fluorescence (TCCF) value

**Fig. 6:**
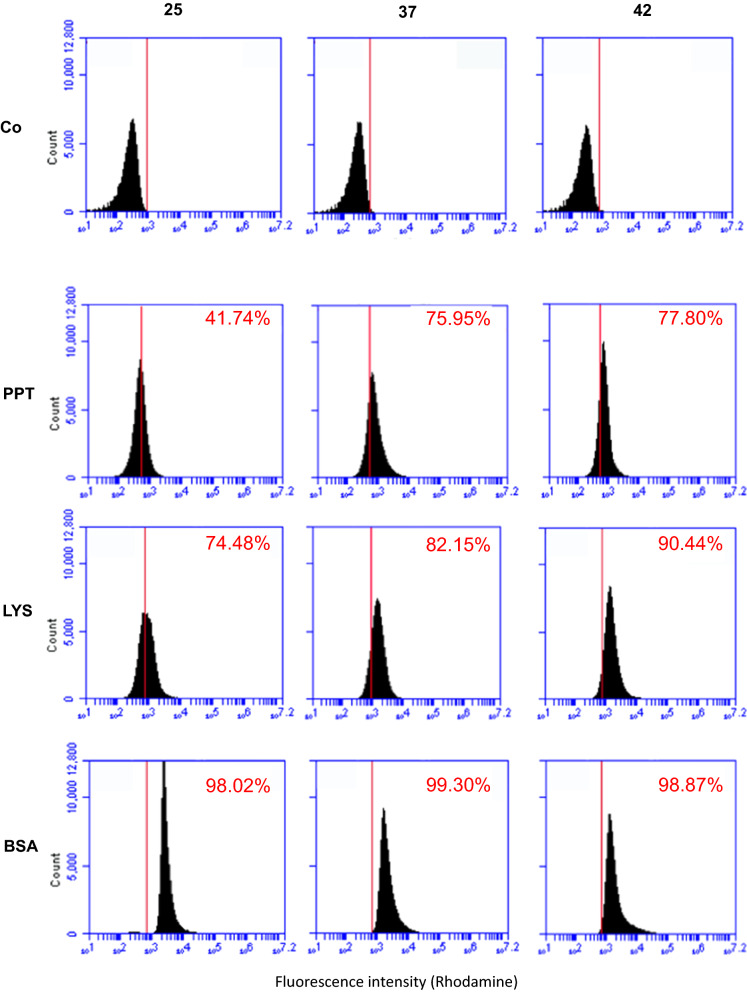
Flow cytometry analysis of spores adsorbed with rhodamine-labeled PPT, LYS or BSA. Free spores (Co) and spores adsorbed with rhodamine-labeled PPT, LYS or BSA were analyzed with a Flow Cytometer. In each panel is indicated the percentage of positive events above the Rhodamine-fluorescent intensity threshold of 1 × 10^3^ (red line)

To evaluate the effects of the temperature on non-recombinant spore-display (adsorption) we used three model proteins: the pentapeptide HPHGH (herein PPT) of 0.77 kDa [26], the commercially available lysozyme (herein LYS) of 14.4 kDa (Sigma) and the commercially available bovine serum albumin (herein BSA) of 66.4 kDa (New England-Biolabs). All three proteins were fluorescently labeled with rhodamine as previously described [26] and 10 mM of each model protein independently used for adsorption with 5.0 × 10^8^ purified spores of the *B. subtilis* strains PY79 [27] produced at 25, 37 or 42 °C. The adsorption reactions were carried out for 1 h at 25 °C in 50 mM Sodium Citrate buffer, pH 4.0, as previously described [11]. Adsorbed spores were collected by centrifugation and analysed by fluorescence microscopy and flow cytofluorimetry, as previously described [24]. As shown in Fig. 5, all three proteins were adsorbed to the spores and the fluorescent signal distributed all around the spore surface. The relative fluorescence signals were analyzed by the ImageJ software (NIH), as previously reported [24]. Since the proteins were fluorescently tagged with rhodamine, an amine-specific label, the number of fluorophore molecules attached to each protein was different, impairing a comparison of fluorescence levels between different proteins. However, the analysis allowed to conclude that: (i) PPT adsorbed with similar efficiency to 37 °C and 42 °C and slightly less efficiently to 25 °C spores (37 = 42 > 25); ii) LYS had a pattern of adsorption similar to that described for PPT (37 = 42 > 25); and (iii) BSA adsorbed at similar levels to 25, 37 or 42 °C spores (25 = 37 = 42) (Fig. 5). Adsorbed spores were analyzed by flow cytometry and the percentage of positive-fluorescent events was obtained as described for Fig. 2. This quantitative analysis performed in duplicate on 100,000 spores/each, confirmed the fluorescence microscopy results of Fig. 5, indicating that PPT was absorbed much more efficiently at 37 or 42 °C, with respectively 75.95 and 77.80% positive events (p.e.) than at 25 °C (41.74% p. e.) (Fig. 6). A similar trend was observed with LYS, although the differences were smaller with 74.48, 82.15 and 90.44% p.e. at 25, 37 and 42 °C respectively, while no differences were observed with BSA with spores prepared at the three temperatures (Fig. 6).

Although the molecular mechanism of spore adsorption is not known in detail, it is likely that more factors are involved in the process. The negative electric charge and relative hydrophobicity of the spore surface have both been shown to influence the efficiency of adsorption [10, 14]. Since it has been previously reported that 25 °C spores are more hydrophobic than 37 and 42 °C spores [22], we hypothesized that the different relative hydrophobicity of spores could explain the reduced efficiency of adsorption of PPT and LYS to 25 °C spores. However, the GRAVY value, an estimation of protein hydrophobicity calculated by adding the hydropathy values of each amino acid residue of a protein and dividing by the number of residues in the protein [28], for PPT, LYS and BSA were − 2.32, − 0.15 and − 0.45, respectively, with increasing positive values indicating an increasing hydrophobicity. Therefore, proteins with the least (PPT) and the highest (LYS) hydrophobicity value showed a similar adsorption pattern (Figs. 5,6), making it unlikely that the hydrophobicity is a major determinant of the efficiency of adsorption, in our experiments. Other physical and chemical parameters of the heterologous proteins, including probably the size and the isoelectric point, have to be considered as they may mediate the ability of proteins to cross the outermost spore layers [15–17], resulting in relevant for the efficiency of the process.

### Localization of proteins adsorbed on 25, 37 or 42 °C spores

A previous report showed that RFP when adsorbed to spores is able to cross the crust and the outer coat, localizing at the inner coat level [15]. In that study, the RFP fluorescence signal was localized by comparison with the signal due to GFP fused to proteins known to be localized in various spore coat layers [15]. A similar approach was used to evaluate whether the temperature of spore production also affected the localization of the adsorbed proteins within the coat. Since the high red fluorescence signal produced by rhodamine-labeled PPT, LYS or BSA overlapped (and caused interference) with the region of detection for the GFP signal, the localization assays were performed adsorbing RFP to spores carrying the *cotC:: gfp* fusion [15] and prepared at 25, 37 or 42 °C.

**Fig. 7:**
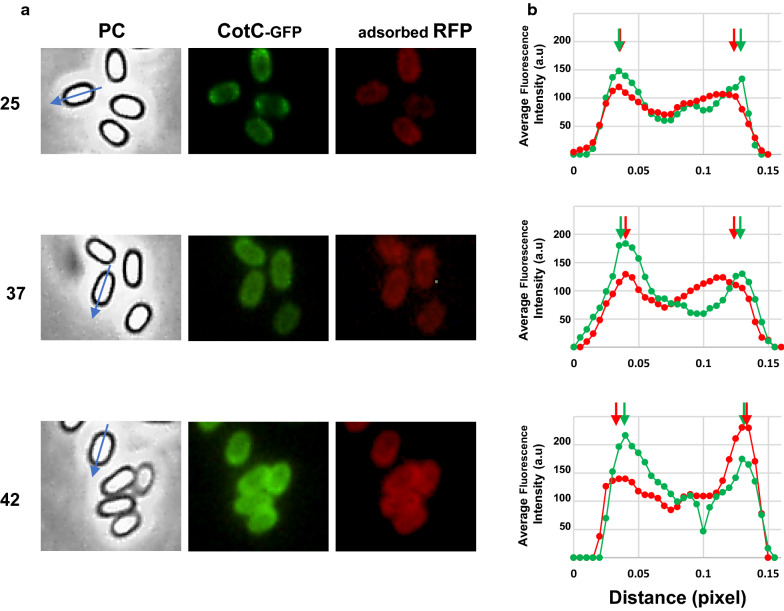
Fluorescence localization of adsorbed-RFP. **a** Spores carrying the *cotC::gfp* were produced at 25, 37 or 42 °C, adsorbed with RFP, and observed by fluorescence microscopy. For each condition, the same microscopy fields were observed by phase contrast and fluorescence microscopy (green and red). A blue arrow in each phase-contrast panel (PC) indicates the long axis of the spore used to plot red and green fluorescence intensities. **b** plots of mean green and red fluorescence intensities along the long axis of 80 different spores. Green and red arrows indicate peaks of GFP and RFP fluorescence intensities, respectively. One pixel corresponds to 1.18 nm. *a.u.* arbitrary unit

As previously reported [15], in 37 °C spores the red fluorescence signal of RFP was internal to the green signal of CotC-GFP (Fig. 7). While RFP localization did not change with 25 °C spores, it was slightly altered with 42 °C spores where the RFP signal was external with respect to the CotC-GFP signal (Fig. 7). The different localization of RFP is most likely due to the different coat structure of spore produced at the various temperatures and indicates that the lamellar and highly electron-dense outer coat (CotB-CotG rich) produced at low temperatures [22] is somehow a more permeable than the granular and thick coat (CotC rich) produced at 42 °C [22], at least with respect to RFP.

## Conclusions

Main conclusion of this study is that the temperature of spore production affects the display of heterologous proteins on the spore surface:

- with the recombinant display the temperature modulates the amount and the surface exposure of the displayed proteins with CotB- and CotG-based fusions more efficient at low temperatures and CotC-based fusions are more efficient at high temperatures;
- when a recombinant spore carries two heterologous proteins each of them is differentially displayed at different temperatures on the base of the carrier used;
- with the non-recombinant display a modest effect is observed with small proteins (PPT and LYS) adsorbed more efficiently by 37 or 42 °C spores than by 25 °C spores;
- the localization of adsorbed RFP within the spore surface layers is modified by the temperature, indicating that spores produced at the low temperatures (CotB/CotG type coat) or at high temperature (CotC type coat) [22] have different adsorption properties.

Overall, this study indicates that the temperature of spore production is an essential parameter to be considered in the development of a spore-display system.

## Materials and methods

### Spore production, extraction of coat proteins and western blot analysis

Sporulation at 25, 37 and 42 °C was induced by the exhaustion method in Difco Sporulation (DS) medium as recently reported [24, 29]. Mature spores were purified by cold-water washing using overnight incubation in H_2_O at 4 °C to lyse residual sporangial cells. Spore purity (higher than 95%) was checked under optical microscope.

Spore coat proteins were extracted from a suspension of spores by SDS-DTT or NaOH treatment [30]. The concentration of extracted proteins was determined by using Bio-Rad DC protein assay kit (Bio-Rad), and 20 μg of total spore coat proteins were fractionated on 12.5% SDS polyacrylamide gels and staining by Brilliant Blue Coomassie or electro-transferred to nitrocellulose filters (Bio-Rad) for western blot analysis following standard procedures. CotC- and CotB- substrate specific antibodies were used at working dilutions 1:7000 for CotC-TTFC and CotB-TTFC detection [5, 21]. Then, a horseradish peroxidase-conjugated antirabbit secondary antibody was used (Santa Cruz). Western blot filters were visualized by the electro chemi luminescence method as specified by the manufacturer and processed to improve the contrast level using ChemidocXRS software (Bio-Rad).

The experiments have been repeated twice analyzing two distinct coat protein extractions.

### Labeling with Rhodamine

2 mg/ml of pentapeptide HPHGH (PPT), commercially available lysozyme (LYS-Sigma), and bovine serum albumin (BSA-New England-Biolabs) were labeled with 50 µl of Rhodamine B isothiocyanate (Sigma) (1 mg/ml in DMSO) as specified by the manufacturer. The protocol is based on the reaction between the isothiocyanate group of Rhodamine and epsilon-NH_2_ of Lysine residues of the protein to be labeled in order to obtain a fluorescent complex. Final molar Rhodamine/Proteins ratio was 0.06 and the labeling reactions were performed pH 8.5. The labeling was followed by dialysis in 1×PBS to remove the unbound fluorescent excess and lyophilization.

### Binding reaction

10 mM of PPT-Rd, LYS-Rd, BSA-Rd were added to a suspension of 5.0 × 10^8^ wild type spores, produced at different temperatures, in 50 mM sodium citrate pH 4.5 in a final volume of 200 µl. For the reaction with RFP, 1 µg of purified protein was added to the suspension of 1.0 × 10^8^ spores produced at different temperatures, in 1.5 M PBS pH 4.0 in a final volume of 200 µl. After 1 h of incubation at 25 °C, the binding mixtures were washed and centrifuged (10 min at 13,000*g*) to fractionate adsorbed spores (pellet) from unbound protein (supernatant).

### Flow cytometry

Recombinant spores expressing TTFC were analyzed by flow cytometry as previously described [31]. Briefly, 10^6^ purified spores were incubated at room temperature for 30 min at room temperature in phosphate-buffered saline (PBS)-3% fetal bovine serum (FBS) prior to 1 h-incubation with anti-TTFC polyclonal antibodies diluted at 1:20 in 1×PBS–1%FBS. After three washes in 1×PBS, fluorescein isothiscyanate (FITC)-conjugated anti-rabbit immunoglobulin G (1:64; Sigma) was added and the mixture was incubated for 1 h at room temperature, followed by four washes in PBS.

For spores adsorbed with PPT-Rd, LYS-Rd and BSA-Rd, a total of 10^6^ spores were resuspended in 0.5 ml of binding buffer and directly analyzed.

Flow cytometry analysis was performed by BD Accuri™ C6 Cytometer and BD Accuri™ C6 Software (BD Biosciences, Inc., Milan, Italy) collecting 100,000 events. Spore without the addition of primary and secondary antibodies or not adsorbed were used to measure the unspecific fluorescence, allowing to set the threshold of positive events at 1 × 10^3^ fluorescence intensity. The experiments were repeated twice analyzing two independently prepared samples.

### Fluorescence microscopy

10^5^ adsorbed spores were resuspended in 50 µl of binding buffer and observed with an Olympus BX51 fluorescence microscope fitted with a 100 × objective UPlanF1 and U-MNG or U-MWIBBP cube-filters to detect the red and green fluorescence emission respectively. The exposure times are in the range between 500 and 1000 ms. Captured images were processed with Image Analysis Software (Olympus) for minor adjustments of brightness, contrast and color balance and for creation of merge images. For RFP adsorbed spores, the fluorescence intensities and the distance between two fluorescent peaks were measured using unadjusted merged images with Image J processing software (version 1.48, NIH) as previously described [15]. To obtain the total corrected cellular fluorescence (TCCF), an outline was drawn around several fluorescent spores and area, integrated density and the mean fluorescence measured, along with several adjacent background readings. The TCCF was calculated by subtracting the area of selected cell × mean fluorescence of background readings to the integrated density.

## Data Availability

All data generated or analysed during this study are included in this published article and are available from the corresponding author on reasonable request.
